# Synchronous Bilateral Solid Papillary Carcinomas of the Breast

**DOI:** 10.1155/2013/812129

**Published:** 2013-06-17

**Authors:** Noriko Yoshimura, Shigeru Murakami, Mayumi Kaneko, Akio Sakatani, Naoki Hirabayashi, Wataru Takiyama

**Affiliations:** ^1^Department of Surgery, Hiroshima City Asa Hospital, Hiroshima 731-0293, Japan; ^2^Department of Pathology, Hiroshima City Asa Hospital, Hiroshima 731-0293, Japan

## Abstract

We herein report a case of synchronous bilateral solid papillary carcinoma of the breast. A 73-year-old female had a mass that was detected in the right breast on mammography. An ultrasound examination revealed one intracystic tumor in the right breast and two tumors in the left breast. A fine-needle aspiration biopsy of these three tumors was performed, which revealed a diagnosis of malignancy. A magnetic resonance imaging examination of the breasts showed diffuse small nodules surrounding these tumors bilaterally. Bilateral partial mastectomy and a sentinel lymph node biopsy were performed. Lymph node metastasis was detected in the right axilla, and additional lymph node dissection was performed. The pathological diagnosis was synchronous bilateral breast cancer, invasive ductal carcinoma NOS of the right breast, mucinous carcinomas of the left breast, and bilateral SPCs. A wide range of surgical margins were positive for SPCs, and additional bilateral total mastectomy was then performed. To the best of our knowledge, little is known about synchronous bilateral SPCs. Our case indicates that some SPCs can be widely scattered and make up a variety of invasive carcinomas. It is difficult to make a correct preoperative evaluation in such cases.

## 1. Introduction

Solid papillary carcinoma (SPC)is a special type of carcinoma that accounts for 1.1–1.7% of all cases of breast cancer [1–5]. It is a malignancy with low-grade nuclear atypia that develops predominantly in elderly patients and clinically behaves as a mass-forming *in situ* carcinoma. SPC is also known to infrequently involve some types of invasive ductal carcinoma, especially mucinous carcinoma. We herein report a case of synchronous bilateral solid papillary carcinoma of the breast.

## 2. Case Report

**Figure 1:**
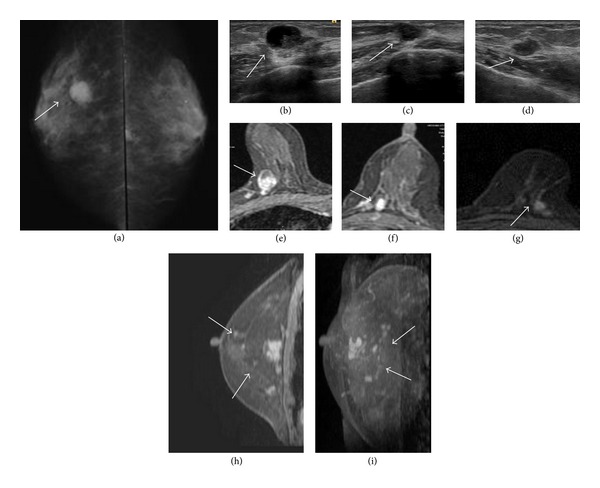
Mammogram showing a round, high-density mass in the right breast ((a), arrow). Ultrasound view showing tumor 1 in the right breast (b) and tumors 2 and 3 in the left breast ((c), (d), resp.; arrows). Magnetic resonance imaging (MRI) scans showing all of these tumors ((e), (f), and (g); arrows) with diffuse nodules surrounding the tumors in the bilateral breast ((h): left; (i): right; arrows).

A 73-year-old female presented with a mass in the upper inner quadrant of the right breast. Anamnesis and the patient's family history were not appreciable. The tumor was mobile without evidence of dermal invasion, and the axillary lymph nodes were impalpable. A round, high-density mass measuring 17 mm in diameter (tumor 1) was found in the right breast on a mammogram (Figure 1(a)). An ultrasound examination revealed one intracystic tumor in the right breast (Figure 1(b)) and two tumors (each 8 mm in diameter) in the left breast (Figures 1(c) and 1(d), resp.). A magnetic resonance imaging examination also showed these tumors (Figures 1(e), 1(f), and 1(g), resp.) with diffuse small nodules surrounding the tumors in the bilateral breasts (Figures 1(h), 1(i)). The serum CEA and CA15-3 levels were not elevated. A fine-needle aspiration biopsy of the tumors was performed, which revealed a diagnosis of histopathology suspected invasive carcinoma. Bilateral partial mastectomy and a sentinel lymph node biopsy were performed. Lymph node metastasis was detected in the right axilla, and lymph node dissection of the right axilla was performed. Tumor 1 was diagnosed as invasive ductal carcinoma NOS, and tumors 2 and 3 were diagnosed as mucinous carcinoma. All of these invasive tumors involved intraductal SPCs, and a wide range of surgical margins were positive for SPC. Therefore, an additional bilateral total mastectomy was performed on day 21 after surgery. Synchronous bilateral invasive breast cancer with intraductal SPCs was eventually diagnosed. The patient continues to receive oral aromatase inhibitor treatment without recurrence five years after undergoing surgery.

### 2.1. Histopathological Findings

**Figure 2:**
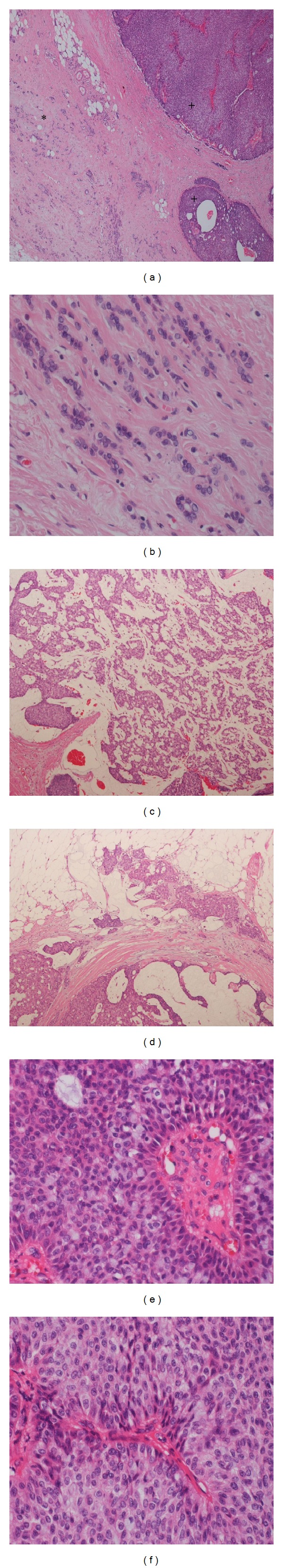
(a) Tumor 1 and the adjacent SPC. The SPC is shown on the upper half (cross), while invasive ductal carcinoma is shown on the lower half (star) (hematoxylin-eosin, original magnification ×10). (b) Tumor 1 was diagnosed as invasive ductal carcinoma NOS. Small alveolar tumor cells structured in a linear growth pattern with fibrosis were observed (hematoxylin-eosin, original magnification ×40). (c) and (d) Tumors 2 and 3 were diagnosed as mucinous carcinomas with tumor cells suspended in abundant cytoplasm mucin (hematoxylin-eosin, original magnification ×10). (e) and (f) SPCs surrounding the bilateral breast. Palisading of tumor cells was evident around the fibrovascular cores. The tumor cells consisted of solid masses of polygonal tumor cells with inconspicuous fibrovascular structures. Extracellular mucin is shown (in the lower portion of 2(e)). The nuclei were small and low grade. Cytoplasmic vacuolization was variably present. Mucicarmine staining demonstrated mucin in the gland lumens and cytoplasm of the tumor cells (hematoxylin-eosin, original magnification ×40).

Microscopically, tumor 1 was diagnosed as invasive ductal carcinoma NOS. Small alveolar tumor cells structured in a linear growth pattern with fibrosis were observed (Figures 2(a)∗ and 2(b)). E-Cadherin staining of the tumor was positive. Tumors 2 and 3 were diagnosed as mucinous carcinomas with tumor cells suspended in abundant cytoplasm mucin (Figures 2(b) and 2(c)). Intraductal SPC components were widely scattered over a range of specimens in the bilateral breasts. Palisading of tumor cells was evident around the fibrovascular cores. The tumor cells consisted of solid masses of polygonal tumor cells with inconspicuous fibrovascular structures. Extracellular mucin was observed. The nuclei were small and low grade. Cytoplasmic vacuolization was variably present (Figures 2(a)+, 2(e), and 2(f)). Mucicarmine staining demonstrated mucin in the gland lumens and cytoplasm of the tumor cells. Immunohistochemically, the SPCs were positive for neuroendocrine markers (chromogranin A and synaptophysin). All of the invasive tumors were positive for estrogen receptor and progesterone receptor but negative for HER2. The Ki-67 labeling indices were tumor 1: 2%; tumors 2 and 3: 8% and 3%, respectively. Additional mastectomy specimens included diffuse intraductal SPCs in the remaining bilateral breasts.

## 3. Discussion

There is a major point for our case. We would like to emphasize that nearly 95% of SPC cases are unilateral [6], and the association between bilaterality and SPC has not been thoroughly discussed. To the best of our knowledge, little is known about synchronous bilaterally extended SPCs, as occurred in our case.

 Generally, SPC is an uncommon type of breast cancer that primarily affects elderly females, with a mean age of 72 years in one series [2–8]. This tumor is characterized by round, well-defined nodules composed of low-grade ductal cells separated by fibrovascular cores. It is currently considered to be an *in situ* carcinoma according to the most recent World Health Organization classification, although, the lack of myoepithelial cells in the periphery of the tumors is intriguing. Almost half of cases are associated with invasive carcinoma and the invasive component may consist of pure mucinous carcinoma, invasive ductal carcinoma, neuroendocrine-like carcinoma, or rarely lobular or tubular carcinoma [1, 2, 5, 7, 9, 10]. Most SPCs are known to be positive for both the ER and PgR receptors, neuroendocrine markers such as chromogranin A, synaptophysin [2–5], and E-cadherin [2]. Conducting immunochemical evaluations helps in making a diagnosis. Due to its low-grade malignant potential, pure SPC has a good prognosis; however, the prognoses of cases associated with invasive carcinoma depend on the invasive carcinoma component [2, 9].

With regard to the differential diagnosis, the microscopic morphology ranges from benign to malignant lesions, including atypical ductal hyperplasia, lobular neoplasia, intracystic papillary carcinoma, and DICS [6]. Atypical ductal hyperplasia does not present with fibrovascular cores. Lobular neoplasia is characterized by discohesion and a lack of papillary fronds. Intracystic papillary carcinoma is characterized by the presence of papillary fronds lined by cuboidal cells that often reveal higher nuclear-grade cytology. DICS, including neuroendocrine DCIS, does not have the monotonous morphology of SPC or cells with a plasmacytoid or spindle cell appearance [11, 12]. Likewise, the presence of mucin, branching fibrovascular stroma, and ducts encompassed by fibrosis are not features of DICS. In our case, making a preoperative diagnosis of SPC was beyond consideration, and we were unable to take into account the surgical margin. Our case indicates that some SPCs can be widely scattered and make up a variety of invasive carcinomas. It is difficult to make a correct preoperative evaluation and perform prompt resection in such cases.

In conclusion, we herein reported a case of synchronous bilateral SPCs. This case demonstrates the unusual clinical behavior of SPC. Further investigation is required to establish optimal management of this malignancy.
